# Supplementary material for: Reducing Biofilm Infections in Burn Patients’ Wounds and Biofilms on Surfaces in Hospitals, Medical Facilities and Medical Equipment to Improve Burn Care: A Systematic Review
Source: Int J Environ Res Public Health. 2021 Dec 14;18(24):13195. doi: 10.3390/ijerph182413195 (PMC8702030; doi:10.3390/ijerph182413195)
Supplement: Supplementary file 1 [file ijerph-18-13195-s001.zip › ijerph-1479265-supplementary.pdf]

International Journal of
Environmental Research and Public Health

Supplemental Table S1. Interventions to reduce infections in burns

Author, date, country	Samples or model	Intervention	Outcome
**Silver compounds**			
Gholamrezazadeh 2018, Iran [6]	28 multidrug-resistant *P. aeruginosa* strains (10^6 cfu/mL) in burns, burn centre, Kerman, Iran; strong biofilm activity (measured by optical density at 480nM) in 28.5% of isolates, 25% moderate, 14% weak, 32% no biofilms. Strains were resistant to ≥ 3 antibiotic classes (ciprofloxacin, aminoglycosides, piperacillin/tazobactam, amikacin, 3rd generation cephalosporins) and 73% sensitive to meropenem and imipenem	Quaternary ammonium disinfectants (Benzalkonium chloride or Deconex) or nano-Ag or nano-Cu	**In vitro** Benzalkonium chloride inhibited growth of all *P. aeruginosa* isolates at MIC 0.06 ± 0.2mg/mL, Deconex MIC 0.5 ± 0.2mg/ml, nano-Ag MIC 20 ± 0.2mg/mL. Nano-Cu had no antimicrobial activity. **In vitro biofilms** (1) Biofilm growth inhibited by Benzalkonium chloride of all *P. aeruginosa* isolates at MBC 0.1 ± 0.2mg/mL, Deconex MIC 1.0 ± 0.2mg/L, nano-silver MBC 28.3 ± 2mg/ml. (2) Biofilm (bacteria 10^6 cfu/ml): nano-Ag (12.5mg/mL) reducted number of *P. aeruginosa* bacteria forming biofilms from 28.5% to 3.5% and benzalkonium (MIC at 0.03 mg/mL) number of bacteria forming biofilms from 28.5% to 18.7%. Nano-Ag (MIC 6.25 mg/mL) reduced cell adherence 7-fold with. **Gene expression** *rhlR* gene expression increased 2-fold with Ag, 7-fold with deconex and 64-fold with benzalkonium
Pourhajibagher 2020, Iran 14]	(1) 100 *A. baumanii* (ATCC 19606) isolates from burn patients in Tehran, Iran. Biofilm inhibition assessed by staining with crystal violet and measuring optical density at 570 nM (2) 40 female BALB/c mice 8-10 weeks of age, 3rd degree burns. *A. baumanii* (1.0 - 2.0 x 10^5 cfu/ml) smeared on wounds	AgSD (silver sulfadiazine); or AgSD-NLs (silver sulfadiazine nanoliposomes); or AgSD-NLs@Cur (silver sulfadiazine nanoliposomes with Curcumin) and all with light emitting diode (LED); or control (no treatment)	**In vitro** (1) MIC for *A. baumanii* isolates from burn patients when exposed to AgSD-NLs@Cur was 15.6 μg/ml; MIC for AgSD-NLs 62.5 μg/ml; MIC for AgSD 125 μg/ml (2) with exposure to MIC_{90} dose of AgSD-NLs@Cur with LED decrease of 99.9% in cell count, AgSD-NLs with LED 68.2% and AgSD with LED 57.3% compared to control. Without LED reductions were 63.4%, 51.7%, and 42.2%. **In vitro biofilms** (1) With MIC_{90} doses of AgSD-NLs@Cur with LED *A. baumanii* numbers decreased by 76.4%, with AgSD-NLs by 44.8% and with AgSD 38.1%. (2) Curcumin docked with active sites of acyl-homoserine-lactone synthase (which promotes biofilm formation) **Gene expression** *luxL* gene expression was reduced 6.7-fold with 1/8 MIC90 of AgSD-NLs@Cur with LED and with AgSD-NLs without LED 2 fold. In vivo With staining and light microsopy untreated mouse burn wounds showed complete loss of the epidermis and hair follicles, hyperemic vessels and extensive bacterial colonization, but those treated with AgSD-NLs@Cur showed focal

			epidermis regeneration, fibrosis and granulation tissue formation.<br>**Cytotoxic effects**<br>No cytotoxic or hemolytic effects observed. Normal human skin fibroblast cells (MHFB-1; IBRC C11179) showed 87.5% survivability
Halstead 2015, UK [13]	*Pseudomonas aeruginosa* 15692 and 1586 from burn wounds; *Acetinobacter baumanii* ATCC BAA-1701 from human blood and 721 burn isolates from Queen Elizabeth Hospital, Birmingham, UK. Biofilms assessed by crystal violet staining at an optical density of 600 nM	Comparison of 11 antimicrobial medicated dressings to prevent biofilm formation by key burn wound pathogens; most tests 8 replications	**In vitro biofilm**<br>Reduction after 72 hours: (a) *A. baumanii 1701* treated with Acticoat 96%; Mepilex Ag 95.9%; acetic acid (concentrations 0.31% to 5%) 90-93%: *A. baumanii 721* treated with Acticoat 100%; Mepilex Ag 100%; acetic acid 5% (concentrations 0.31% to 5%) 90-93% compared to control: (b) *P. aeruginosa* 15692 treated with Acticoat 100%; Mepilex Ag 100%; acetic acid (concentrations 0.31% to 5%) 86-96%; *P. aeruginosa* 1586 treated with Acticoat 94%; Mepilex Ag 99.9%; acetic acid (concentrations 0.31% to 5%) 88-97%.
**Other metal compounds**			
Karaky 2020, UK [8]	*P. aeruginosa* (NK-1 and NK-2) (1 .0 x 10^8 cfu/mL) from patient wounds (n not stated) at Royal Bolton Hospital, UK	18 metal ions in combinations with Graphene or Graphene oxide tested in 96-well microplate. MICs were assessed as high (≤ 30 mg/L); good (≥ 31 mg/L to ≤ 60 mg/L); moderate (≥ 61 mg/L to ≤ 90 mg/L) and no efficacy (≥ 91 mg/L)	**In vitro** (1) MICs: Five metal ions had high MICs against both *P. aeruginosa* strains: platinum (7.8 mg/L), palladium (13 mg/L), tin (13 mg/L), molybdenum (15.6 mg/L) and gold (26 mg/L), rhenium and gallium (31.3 g/L) rhodium (41.7-52.1 mg/L), and aluminum (41.7 mg/L) showed good bactericidal activity<br>(2) MBICs: platinum (7 mg/L) and tin 13.0 mg/L) had high MBICs, rhenium and molybdenum (31.1 mg./L) and gold and palladium (41.7 mg/L good bactericidal activity<br>In vitro biofilms<br>(1) Graphene reduced biofilm forms of the bacteria significantly more than the planktonic forms ($p < .0001$)<br>(2) Eight metal-graphene combinations reduced the amount of intact biofilm by ≥ 90% or more (platinum-graphene oxide, gallium-graphene oxide, molybdenum- graphene oxide, gold-graphene oxide, silver-graphene, gallium-graphene and molybdenum-graphene.<br>(3) The Most reduction of *P. aeruginosa* biofilm metabolic activity occurred with gold-graphene oxide (94%), molybdenum- graphene oxide (93%), silver (91%) and silver-graphene (91%).
Li 2020, China [15]	Burns created on 32 BALB/c mice MRSA (10^7 cfu/mL) applied to wound and also 24 hours later. (No statement of source of *S. aureus* or MRSA)	Bimetallic nanoenzymes were tested for the effect of their peroxidase generation. Wounds treated twice daily for 3 days with H_20_2 and $CuCo_2S_4$ nanoparticles (100µg/mL), or H_20_2, nanoparticles, or no therapy	**In vitro** (1) After treatment with H_20_2 (2nM) and $CuCo_2S_4$ nanoparticles (100µg/mL) after one hour 3.6 log reduction in viability of MRSA, 3.3 log in *S. aureus* and 4.7 log reduction in *E. coli.* H_20_2 and $CuCo_2S_4$ nanoparticles (100µg/mL) separately showed no biocidal activity.<br>**In vitro biofilms** On confocal scanning electron microscopy most of the MRSA cells collapsed and lost their cellular integrity. **In vivo**

			Mouse burns: after 2 days treatment with H_2O_2 (2nM) and $CuCo_2S_4$ nanoparticles (100 μg/mL) no inflammatory response and burn wound contracted. Control group showed severe inflammatory response with suppuration. After 6 days group treated with H_2O_2 (2nM) and $CuCo_2S_4$ showed enhanced healing and 83.7% wound closure; $CuCo_2S_4$ 71%, H_2O_2 63.3%, control 59%. At 2 weeks H_2O_2 (2nM) and $CuCo_2S_4$ wounds completely closed and healed.
Nozari 2021, Iran [16]	*(1) S. aureus* ATCC 25923 and *P. aeruginosa* ATCC 27853<br>(2) four female rats weight 200 g and burn generated 1.5 cm^2	Chitosan-alginate-gelatin, or chitosan-bentonite-ZnO gelatin films and nanoparticles	**In vitro after 18 hours**<br>(1) *S. aureus* with chitosan-alginate-gelatin film for 3 samples ranged from $1x10^4$ cfu/ml to 3.2 x 10^5 cfu/ml compared to control (1.5 x 10^9 cfu/ml); with chitosan-bentonite-gelatin film ranged from 7.8 x 10^5 to 3 x 10^6 cfu/ml compared to control (1.5 x 10^9 cfu/ml), (99.99% reductions). (2) *P. aeruginosa* with alginate film $1x10^4$ to 8.2 x 10^5 cfu/ml compared to control (1.5 x 10^9); with bentonite film 1 x 10^4 to 1.9 x 10^5 cfu/ml compared to control (1.5 x 10^9 cfu/ml), (99.99% reductions). **In vivo:** mouse burns at 7 days: in treated rats re-epithelialisation, active fibroblasts, hair follicles and sebaceous glands were detected. No re-epithelialisation in untreated rats
		**Disinfectants**	
Halstead 2015, UK [17]	29 isolates of common wound pathogens tested (9 *P. aeruginosa*, 8 *Acinetobacter baumanii*, 3 *Escherichia coli*, 3 *Staphylococcus aureus*, 3 *Enterobacter cloacae*, 2 *Klebsiella pneumoniae*) at 10^5 cfu/ml. Biofilm formation assessed by crystal violet staining	Acetic acid at concentrations 0.16% to 5%, duplicate tests.	**In vitro planktonic growth**<br>1. MIC = 0.16% for 9 isolates acetic acid, MIC = 0.31% for 20 isolates **In vitro biofilms**<br>For 23 isolates acetic acid MBIC = 0.31% and MBEC against formed biofilms ranged from ≤ 0.10% to 2.5%; eradication of mature biofilms observed for all isolates after 3 hours exposure.
Song 2016, China [18]	Mice deep second-degree burns, then infected with MRSA ATCC 252 (cfu/mL not stated) Biofilms assessed by OD at 570 nM	Chlorhexidene acetate nanoe mulsion (CNE) (2 mg/mL) compared to Chlorhexidene acetate water solution (CHX) (5 mg/mL)	**In vitro** (1) MICs against MRSA (ATCC 252): CNE MIC was lowest at 1μg/mL, CHX 4μg/mL, benzalkonium 5 μg/mL, povidone iodine 500 μg/mL<br>(2) **Reduced bacterial viability:** 90% by CNE at 8μg/mL within 5 minutes and bacteria completely killed with 8μg/mL by 1440 minutes. CHX at 8μg/mL reduced bacterial viability by 90% at 240 minutes but bacteria not completely killed by 1440 minutes.<br>**In vivo**<br>Mouse burn wound with CNE (5mg/mL) scab detached from wound 8th day, completely detached 29th day; CHX 13th and 33rd days<br>**In vivo biofilms** On scanning electron microscopy MRSA biofilms treated with CNE (2μg/mL) biofilms were "dispersed and disrupted and obvious reduction in number of bacteria" and large vacuoles between cell wall and cytoplasm, and DNA, protein, K^+ and Mg^{2+} leakage; cells

			treated with CHX (2μg/mL) slight inhibition, superficial bacteria deformed, biofilm intact. Dead/live cell ratio with CNE 83.6%, with CHX 13%
Tiwari 2018, India [19]	89 isolates of *S. aureus* from pus exudates from wounds (origins not stated) from which random selection was made of 10 strong biofilm formers (defined as optical density > 4 OD_c) 10 weak (OD from OD_c to 2 OD_c). The strong biofilm group included cellulitis, foot ulcers, osteomyelitis, folliculitis, lymph node discharge, arthritis and 2 burns)	0.6% sodium hypochlorite compared to 70% ethanol, triplicate tests	**In vitro biofilm % reduction in biofilm optical density (OD):** no significant differences comparing reductions of strong and weak biofilm formers for either sodium hypochlorite or ethanol: (1) with 0.6% sodium hypochlorite for strong biofilms 34.3% ± 15.30; and weak biofilms 35.1 ± 13.0 (p = .897); (2) with 70% ethanol for strong biofilms 18.1% ± 11.6 and for weak biofilms 20% (p = .488) On electron microscopy strong biofilm producers showed significant depressions and irregular craters on their surface
		**Hydrogels**	
Andersson 2021, Sweden [9]	Burns in Göttingen minipigs. *S. aureus* or *P. aeruginosa* 30 μL (10^8 cfu/ml)	Prontosan (polyhexamethylene biguanide (0.1%) and undecylenamidopropyl betaine (0.1%), or levofloxacin applied 2 hours after wound creation	**In vivo:** (assessed by visual inspection of a,uthors' Figures 4 and 6)<br>***S. Aureus***<br>(1) **wound surface reductions** *S. aureus* from cfu 10^8 to cfu log 10^6 for levofloxacin (2 μg/ml) compared to control cfu increased to log 10^{10} (p <.0001) (2) **wound tissue reductions** to cfu log 10^6 for levofloxacin (2 μg/ml) compared to control cfu log $10^{8.5}$ (p <.001)<br>(3) **wound surface reductions** to cfu log 10^6 for prontosan compared to control cfu log 10^{10} (p <.0001) (4) **wound tissue reductions** to cfy log 10^7 for prontosan compared to control cfu log 10^8 (p <.05)<br>***P. aeruginosa***<br>(1) **wound surface reduction** no change in cfu log 10^8 for levofloxacin (2 μg/ml) but control increased to cfu log 10^{11} (p <.001)<br>(2) **wound tissue reduction** no change cfu log 10^8 for levofloxacin (2 μg/ml) and control cfu log 10^8 (n.s.)<br>(3) **wound surface reduction** from cfu 10^8 to cfu log 10^6 for prontosan compared to increase in control to cfu log 10^{11} (p <.001)<br>(4) **wound tissue reduction** to cfu log $10^7$for prontosan compared to control cfu log $10^{8.5}$ (p <.05) **Biofilms**<br>(1) On scanning electron microscopy both antibacterial treatments "visibly reduced" number of bacterial cells on wound surface and perturbations and bacterial clumping and debris on prontosan-treated.<br>(2) **Bioluminescence** of bioluminescent-prepared *S. aureus* and *P. aeruginosa* at 2 hours significantly reduced by protosan (p <.001 to .0001)
Chhibber 2020, India [20]	Burns on BALB/c mice. then *S. aureus* (43300) 50 μL containing 10^7	Wounds treated with Conventional hydrogel	**In vivo biofilms**<br>(1) **Conventional hydrogel:** 2.8 log_{10} reduction

	cfu/ml applied to wound then groups of 18 mice assigned to each therapy or control	(moxifloxacin 0.5%w/v and carbomer 1% w/v) or Novel hydrogel (same but included Chitosan 5mg/mL and Boswellia gum 0.5% [a local plant in India]); or control (no treatment)	on day 1; 4.2 log_{10} cfu/ml reduction day 2 and wound became sterile (day not stated)<br>(2) **Novel hydrogel:** 3.5 log_{10} cfu/ml reduction on day 1, 4.8 log_{10} cfu/ml reduction day 2 and wound sterile (day not stated)<br>(3) Control 6.9 log_{10} cfu/ml count day 3.<br>(4) At 4 hours complete eradication of MRSA from wounds with conventional and novel hydrogels but MRSA established in control mice. (5) At 24 hours subdued inflammation and signs of healing in treated mice but in control mice loss of epithelium, neutrophils and thick layer of inflammatory cells.<br>(6) **Toxicity:** Mean wound erythema score 0 in mice treated with either hydrogel and 3 in control mice
**Sonotherapy to release reactive oxygen species (ROS)**			
Pourhaji bagher 2021, Iran [21]	Multi-species bacterial biofilms containing *Staphylococcus aureus, Pseudomonas aeruginosa,* and *Acinetobacter baumannii* in brain heart infusion broth and biofilms grown in 96 well microtitreplate	Sonodynamic therapy (SDT) using sonsosensitizer nanoparticles of nano-emodin (N-EMO) to generate reactive oxygen species	**In vitro** (1) For the multi-species bacterial suspension MIC value of N-EMO was $0.15x10^{-4}$ g/L;<br>**In vitro biofilms** (1) To inhibit multi-species bacterial growth the Minimal Biofilm Eradication Concentration (MBEC) value of N-EMO was $2.5x10^{-4}$ g/L; no effect with ultrasound alone<br>(2) **Reduction in the biofilm of multi-species bacterial growth** following SDT at 1/2 MIC of N-EMO was 81.5 %; at 1/16 MBIC 71.0%; and at 1/128 MBEC 57.8; (reductions in Log_{10} CFU/ml 99.9993%, 99.9736%, 99.4760%)<br>**Gene down regulation**<br>After sub-MBIC of N-EMO genes *lasI* downregulated 2.5-fold; *agA* 3.6-fold and *abal* 5.5 fold; after sub-MBEC of N-EMO 3.0, 5.2 and 7.4
**Light therapy to release reactive oxygen species (ROS)**			
Ishiwata 2021, Japan [22]	38 7-8 week-old Sprague-Dawley rats deep burns (4 x 20 $cm^2$20% = total body surface area) then burn infected with *P. aeruginosa* ATCC 27853 1 x 10^8 cfu/ml	Rats allocated to (1) ethanol and ethylene diamine-tetra-acetic acid disodiumsalt and dimethyl sulfoxide sensitizer (PS group) (n=10); or (2) PS + array of 3 x 6 methylene blue 665-nm LED diodes (45mW/cm^2 at 2.5 cms) 3 times daily for 20 minutes x 7 days (aPDT group) (n=14), or (3) no treatment (n=14).	**In vivo**<br>Baseline: 8.9 x10^4 cfu/ml,<br>**Day 0:** post infection: aPDT group no bacteria, control 3.4 x10^8 cfu/ml;<br>**Day 1:** aPDT 3.5 x 10^5 cfu/ml, PS 4.7 x 10^5 cfu/ml indicating rapid regrowth<br>**Days 2-7:** rapid regrowth each day and aPDT group day 6 x 10^4 cfu/ml<br>**Rat survival:** at day 7: aPDT 11/14. PS 3/10, control 2/14
Lu 2021, USA and China [3]	(1) 11 strains of *Acinetobacter baumannii (Ab), Pseudomonas aeruginosa (Pa) and* MRSA (each 7.5 log cfu/ml) (2) full thickness 3rd degree burns in mice (n not stated)	(1) In vitro the phytochemical carvacrol (0.2 mg/ml) and (2) 450 nm blue light (75J/cm^2) to excite porphyrin-like derivatives in bacterial cells to produce reactive oxygen species (ROS) (2) in vivo mice burns infected with *Acinetobacter baumainii* (5 x 10^5	**In vitro**<br>With either blue light or carvacrol 5% on propidium iodide staining cell envelopes of *Ab* damaged 95%, *Pa* 89%, MRSA 90%, and on SEM cytoplasmic vacuoles and stainless vesicles<br>**In vitro biofilms** *Ab, Pa AF0001 and* MRSA IQ0064 biofilms (10^7 cfu/ml) completely eliminated after 22.5 minutes of

		cfu/ml) and 50 µl carvacrol (1 mg/ml)	blue light + carvacrol (p <.0001) and reduced Ab biofilm from 58.6 µm to 1.4 µm thickness and MRSA from 32.4 µm to 1.7 µm; six first-line antibiotics inactivated < 1.5 log cfu/ml after 6 hours **In vivo mouse burns** Carvacrol 50 µl (1mg/ml) + blue light for 12 minutes (40 J/cm^2) with luminescent bacteria eliminated log 8 luminescence, blue light alone 2.3 log, and carvacrol 0.8 log Reactive oxygen species (ROS): increased 14-fold in Ab, 12-fold in PA AF001 and 8-fold in MRSA IQ0064
Pourhaji bagher 2020, Iran [2]	Multi-species bacterial suspension including *Acinetobacter baumannii, Pseudomonas aeruginosa, Staphylococcus aureus* from Iranian Biological Resource Centre, Tehran. brain heart infusion broth.	Photodynamic therapy (aPDT) using photosensitiser indocyanine green (ICG) and diode laser at 810 nm; assays in triplicate. Morphological changes assessed by scanning electron microscopy	**In vitro** (1) **Reduction in cell viability**: ICG (1000 µg/ml) significant reduction in cell viability of *A. baumannii* (1.5 x 10^5 cfu/ml), *P. aeruginosa* (1 x 10^5 cfu/ml); *S. aureus* (1.0 x 10^5 cfu/ml) compared to control (4.5 x 10^5 cfu/ml) (all p <.05). (2) **Increase in production of reactive oxygen species**: 54% compared to controls. (3) **Expression of quorum-sensing genes:** *abal* reduced by 1.9-fold, *agrA* by 3.7-fold and *lasI* by 4.9-fold in *P. aeruginosa* assessed by real time RT-PCR (4) **In vitro cell morphology** By scanning electron microscopy diode laser + ICG reduction in cell size and numbers, cell elongation, increased cell destruction. No change with diode laser or ICG individually
Wang 2016, China [4]	(1) *P. aeruginosa* ATCC 19660 (strain 180) and multidrug resistant clinical strain *of A. baumanii* in 96 well microplates. (2) Female BALB/c mice age 7-8 weeks, 3rd degree burns.	Exposure to light-emitting diode with antimicrobial blue light (aBL) (432J/cm^2) at wavelength 415 nm to excite endogenous porphyrins to release ROS. Assays replicated x 4	**In vitro** (1) exposure of 24-hour-old and 72-hour-old *A. baumanii* biofilms to aBL (432J/cm^2) for 72 minutes resulted in inactivation of 3.59 $\log_{10}$ and 3.18 $\log_{10}$ cfu/ml; (b) exposure of *P. aeruginosa* biofilms to aBL (432J/cm^2) resulted in inactivation of 3.02 $\log_{10}$ and 3.12 $\log_{10}$ cfu/ml. Control biofilms showed <0.27 $\log_{10}$ cfu/ml loss of viability for *A. baumanii* and <0.42 $\log^{10}$ cfu/ml for *P. aeruginosa.* **In vivo:** Mouse burn wounds infected with *A. baumanii* (5 x 10^6 cfu/ml): at 24 hours required 360 J/cm^2, and at 48 hours 540 J/cm^2 to inactivate 3 $\log_{10}$ cfu/ml in biofilms. **In vivo endogenous porphyrins:** were demonstrated in *A. baumanii* and *P. aeruginosa* by high-performance liquid chromatography
		**Small molecules**	
Banar 2016, Iran [23]	57 *P. aeruginosa* samples (31% strong, 47% medium and 22% weak biofilms) from burn wound patients in Iran University of Medical Sciences Hospital, Iran. Optical density of biofilms assessed as weak if ODc < OD <	Treatment with ceftazidime (CAZ) 1024 µg/mL; or CAZ + α-mannosidase 4 µg/mL, or CAZ + β-mannosidase 4-8 µg/mL, or CAZ + trypsin 8-32 µg/mL; all assays in triplicate	**Minimum biofilm eradicating concentration (MBEC):** Strain 1: ceftazidime (CAZ) 1024 µg/mL; CAZ + α-mannosidase 128 µg/mL, CAZ + β-mannosidase 128 µg/mL, CAZ + trypsin 512 µg/mL;

	2xODc; moderate of 2xODc < OD < 4xODc; strong if < 4x ODc; Strains 1-3 that were tested were susceptible to ceftazidime and amikacin in the planktonic state and resistant in the biofilm state and were included in study.		Strain 2: ceftazidime (CAZ) 1024 μg/mL; CAZ + α-mannosidase 4 μg/mL, CAZ + β-mannosidase 4 μg/mL, CAZ + trypsin 8 μg/mL; Strain 3: ceftazidime (CAZ) 1024 μg/mL; CAZ + α-mannosidase 4 μg/mL, CAZ + β-mannosidase 8 μg/mL, CAZ + trypsin 32 μg/mL; All tested concentrations killed biofilm bacterial cells
Ghosh 2015, India [24]	(1) Bacterial samples from Department of Neuro microbiology, National Institute of Health and Neuro Sciences, Bangalore, India. *K. Pneumoniae* ATCC 70063, R3421; *A. baumanii* MTCC 1425, R674, R676; *P. aeruginosa* MTCC 424, R590, R3324; *E. coli* MTCC 443, R250; *E. Cloacae* R2928. (2) The Study also focused on persister cells (cells in stationary phase) which can down regulate their metabolic processes during antibiotic treatment and are responsible for chronic infections 3. Mice (4-5 per test group), no details	(1) Small molecular antimicrobial compounds with an aromatic core (naphalene (N) or benzene (B)), a L-lysine moiety and a variable lipophilic chain. A naphthalene core compound NCK-10 has a decyl chain appendage and is the most active against NDM-1 producing Gram-negative pathogens and was chosen for this study. (2) A benzene core molecule (BCK-12) has similar MICs to NCK-10	**In vitro biofilms** (1) NCK-10 for most isolates MIC 4.5 μg/mL (2) **Persister cells:** NCK-10 completely lysed persister cells of *E. coli* (5 log cfu/ml) after 2 hours but colonies persisted in control group at 5 log cfu/ml. (3) **Disruption of biofilms:** EC_{50} = 30 μM against biofilms of *A. baumanii* (MTCC 1425); 20 μM against *E. coli MTCC 443); 26* μM against *K. pneumoniae (ATCC 700603),* and 19 μM against *P. aeruginosa (MTCC 424).* On confocal microscopy in the treated samples the biofilms were completely disrupted, and the untreated samples had biofilms 12.6 μm thick. (4) NTK-10 did not induce bacterial resistance (no change in MIC after 20 passages), but MIC of colistin increased 250-fold. (5) In whole blood NCK-10 at 10 times MIC reduced *E. coli* from 7.1 log cfu/mL by > 3 log within 3 hours, whereas untreated samples increased to 8.3 log cfu/ml. **In vivo** In burn wounds of mice significant reduction in bacterial burden after daily topical treatments with NCK-10 (40mg/kg) x 7 days compared to control; no colonies observed with colistin (5mg/kg)
Goodwine 2019, USA [1]	(1) 12 *P. aeruginosa* and 6 *S. aureus* strains from wound debridement samples Southwest Regional Wound Clinic, Lubbock Texas; second degree burn wounds; newborns with cystic fibrosis. (2) Second-degree burn wounds in 3 pigs inoculated with *P. aeruginosa*	Biofilms exposed to enzyme pyruvate-dehydrogenase (PDH) to test efficacy of tobramycin killing of wound biofilms. PDH catalyzes pyruvate to acetyl-CoA in the presence of CoA and NAD^+ and the microcolony formation factor MifR.	**In vitro** (1) Samples from human wounds: 2.2 fold reduction after exposure to 5 mU DPH and by 2.9 fold after 10-20 mU; **In vitro biofilms** (1) On confocal laser scanning microscopy 60% of microcolonies in PDH-treated biofilms showed signs of dispersion with central voids, and 8% of untreated biofilms. (2) 4 day old human wound samples of *S. aureus* biofilms exposed to PDH 10 mU had 40% reduction in mass **In vivo** (1) **Pig burn wounds:** *P. aeruginosa* biofilm population mass reduced 2-log with tobramycin (200 μg/mL) compared to untreated control; (2) 4-log reduction by tobramycin (200 μg/mL) + PDH (200mU) compared to control; (3) silver sulfadiazine 2-log reduction in biofilm and 4-log in planktonic populations.

			(4) Silver sulfadiazine denatured PDH so could not be combined.
Han 2018, China [25]	(1) 10 Ten *S. aureus* strains including GIM 1.55, *S. aureus* GIM 1.771 (MRSA) resistant to ciprofloxacin, gentamicin, kamamycin, chloramphenicol, methicillin and tetracycline, and *S. aureus* CICC10790 (origins of samples not stated) (2) Mice with scald wounds (numbers of mice and origin., size and degree of burns not stated)	Li-F peptide AMP-jsa9 is a cyclic lipopepsipeptide antibiotic (isolated from *Paenibacilllus polymyxa)* which penetrates MRSA cell membranes and kills planktonic cells	**In vitro**<br>Passage across cell wall: on confocal laser microscopy: AMP-jsa9 after 60 minutes permeated cell wall and was widely distributed throughout the cytoplasm of the three *S. aureus* strains tested.<br>**Ultrastructural changes:** on atomic force microscopy (AFM) and transmission electron microscopy GIM 1.55 strains showed severe membrane collapse with corrugations and depressions<br>DNA changes on ATM: with AMP-jsa9 DNA showed local changes and aggregation<br>**Biofilms:** Reduction of cell viability of *S. aureus* CICC10790 to 10% with 8 x MIC Vancomycin (8 µg/mL) and to 10% with AMP-jsa9 at 8 x MIC (128µg/mL); reduction of biomass to 15% with 8 x MIC Vancomycin (8 µg/mL) and to 15% with AMP-jsa9 at 8 x MIC (128µg/mL);<br>**In vivo**<br>In mouse scalded skin burns viable cell count treated with vancomycin or AMP-jsa9 were 10^1 to 10^2 on days 3 and 7 and in those treated with kanamycin or saline 2-3 x 10^4 at 3 days and 5-6 x 10^5 at 7 days with a large infiltrate of inflammatory cells
Konai 2020, India [5]	(1) *A. baumanii*-R674 and *P. aeruginosa*-R590 clinical isolates<br>(2) Burns in 20 6-8 week old female BALB/c mice. Randomised to groups of 5 mice injected with *A. baumanii*-R674, or *P. aeruginosa*-R590, or topical antibacterials, or control (no treatment)	D-LANA-14 is a D-lysine conjugated aliphatic norspermidine analogue with a tetradecanoyl chain with the ability to depolarise gram negative cell membranes; 24 hours after wounds were created the antimicrobial burden was $10^{7\text{-}8}$Ccfu/g. Then mice received topical treatments daily x 6 days or D-LANA-14 (40 mg/kg) plus rifampicin (40mg/kg); or colistin (20 mg/kg), or no treatment.	**In vitro** (1) D-LANA-14 depolarised cytoplasmic membrane potentials<br>(2) Against 3 strains of *A. baumanii* and 4 strains of *P. aeruginosa* D-LANA-14 "moderately active" at MICs 32-64 µg/mL; the combination of D-LANA-14 at sub-MIC levels enabled tetracycline (4 µg/mL) and rifampicin (2 µg/mL) to be active against both antibiotic groups. **In vitro biofilms** With confocal scanning electron microscopy D-LANA-14 (8µg/mL) plus colistin (8 µg/mL) resulted in > 80% reduction in biofilm mass of *A. baumanii*-R674 and *P. aeruginosa*-R590; D-LANA-14 (8µg/mL) showed no effect, and rifampicin (8µg/mL) showed 25-30% disruption In vivo **Burn wounds in mice:** D-LANA-14 (40 mg/kg) plus rifampicin (40 mg/kg) caused 4.9 log reduction in *A. baumanii*-R674 and 4.0 log in *P. aeruginosa*-R5902; D-LANA-14 2.3 log and 1.3 log; and rifampicin 3.0 log and 1.6 log.
Memariani 2016, Iran [26]	*P. aeruginosa* strains ATCC 27853 from burn wounds of hospitalised patients in Iran (n not stated) (5 x 10^7 cfu/ml), and MDRPA (multi-drug resistant) strains numbered 1-5 in 96 well microplates	Short hybrid antimicrobial peptide PV3, which includes residues from two snakes: pEM-2 from *Bothrops asper* and Mastoparan-VT-1 from *Vespa*	**In vitro** (1) ***P. aeruginosa:*** ATCC 27853 for PV3 MIC = 1; for 5 strains of MRDPA for PV3 MIC = 2-4; for ATCC 27853 MIC for ceftazidime = 1; for 5 strains of MRDPA MIC = 16-256.

		tropica. (MIC and MBC stated as 2-4 μg/ml)	(2) **Minimal bactericidal concentration (MBC):** for ATCC 27853 MBC PV3 = 1; for 5 strains of MRDPA MIC PVC = 2-4; for ATCC 27853 MIC ceftazidime = 16-256; for 5 strains of MRDPA MIC = 16-256. **In vitro biofilms** Scanning electron microscopy with acridine-orange/ethidium bromide staining: PV3 treated cells were shorter, blisters on membranes, roughness and blebbing. For PV3 at 8 x MIC at 24 hours resulted in "almost" 100% killing of cells and 95% biomass removal.
Pan 2020, China [27]	(1) *P. aeruginosa* and MRSA (origin not stated) grown in 96 well microtiter plate; triplicate plate counts for biofilm killing assay. (2) Burn wounds in female mice 6-8 weeks old (n not stated) from Institute of Cancer Research (ICR). Wounds injected with *P. aeruginosa.*	Amino-acid-based star-shaped poly(L-ornithine)s and poly(L-lysines) with varying surface charge/hydophobicity balances (P03, PL2, PH3) to disrupt bacterial cells and biofilms	**In vitro** (1) P03 at 2 x MIC (9 μg/ml) and PL2 at 2 x MIC (12 μg/ml) eradicated MRSA; P03 at MIC (12 μg/ml) completely killed *P. aeruginosa.* On scanning electron microscopy and transmission electron microscopy PO3 in *P. aeruginosa* (100 μg/ml) exhibited "remarkable distorted and wrinkled cell wall" and cell wall and membrane lysis, compared to controls. **Biofilms** P03 reduced the biomass of *P. aeruginosa* biofilms by 76.9%, PL2 by 35.1% and PH2 by 31.45%, Polymixin by 7.8% **In Vivo** In mice burn wounds: P03 caused 78.2% reduction in *P. aeruginosa* and PL2 caused 49.3% reduction compared to Polymixin B. **Toxicity:** mice received IV 1 dose of P03 or PL2 (5.5 mg/kg^{-1}) and all mice were active and survived without signs of illness and with normal AST, ALT, Cr and urea levels after 7 days
Su 2019, China [28]	(1) *S. aureus* ATCC 2913 and MRSA (local hospitals in central China). (2) Burns in female C57BL/6J mice	Platensimycin (PTM) and Platensimycin-thioether analogues (PTM-2t). Mice infected with 0.5 ml MRSA (2-3 x 10^7 cfu/ml). [Platensimycin targets Fab/FabF of bacterial fatty acid synthases and was discovered by Merck Research laboratories on whole cell screening of 250,000 natural product extracts in *Streptomyces platensis*]	**In vitro biofilms** Microtiter dish biofilm formation assays: after 2 μg/mL PTM or PTM-2t biofilm formation for *S. aureus* ATCC 291213 reduced 95%. **In vivo** Mouse burns treated with 4 mg of PTM or PTM-2t on burn wound twice daily x 7 days. PTM reduced *S. aureus* to 2 x 10^6 cfu/g and PTM-2t to 8.6 x 10^6 cfu/g compared to 2.5 x 10^6 cfu/g for mupirocin and untreated mice 4.3 x 10^8 cfu/g. On hematoxylin and eosin staining untreated mice showed partially destroyed hair follicles, incomplete fat layer, a large number of inflammatory cells in the muscle layer; treated mice had "relatively healed skin structure."
Uusitalo, 2017 Sweden [29]	1. *P. aeruginosa* (ATCC 19660) cultures in vitro 2. Burn wounds on *P. aeruginosa* (ATCC 19660) ten 6-week old female BalB/c mice	Virulence blocker salicylidene acylhydrazide, INP0341, which inhibits translocation of four exoenxyme (Exo) molecules S,T, U and Y from bacterial cytosol directly into host cytoplasm	**In vitro** (1) INP0341 significantly reduced in a dose dependent manner expression and secretion of the Type III secretion system T3SS ExoS by *P. aeruginosa* required for colonisation and survival in host cells.

		where they alter cell function to permit bacterial growth. Mice wounds infected with *P. aeruginosa* (ATCC 19660) (1 x 10^6 cfu/ml) and treated with INP0341 (70 μM) 4x daily x 1 day then twice daily	(2) INP0341 inhibited toxic activity of *P. aeruginosa on* HeLa cells but *P. aeruginosa* without INPO341 showed rounding of the cells In vitro biofilms (1) INP0321 (100 μM) reduced biofilm to 40% of control (p <.05) (2) INP0341 inhibited *P. aeruginosa* swarming and prevented movement across semisolid surfaces which requires flagellae and type IV pili. In vivo Treated mice died at 36 hours, controls as 42 hours (p <.05)
	**Glycans**		
Wheeler 2019, USA [30]	Four female Yorkshire pigs (weight 70-80 lbs), each received six 2″ x 2″ burn wounds on back	*P. aeruginosa* PA01 (1 x 10^5 cfu/ml) injected into wounds. Wounds randomised on days 1 and 4 to treatment with MUC5AC glycans from fresh pig intestines, MUC2 from pig stomachs, and MUCB human salivary glycans (n not stated), or monosaccharides as control	**In vitro** *P. aeruginosa* PA01 biofilms exposed to mucins: (1) 70% of cells dissociated from surface into planktonic phase (p<.0001). (2) intestinal mucins suppressed quorum sensing (*lasR*), siderophore biosynthesis (*pvdA*) and type-three secretion (*pcrV*) genes. (3) MUC5AC and MUC5B (0.5% w/v) suppressed virulence pathways 1, 2, 3 and 6 secretion systems, siderophore biosynthesis, (pyoverdine and pyochelin) and quorum sensing. (4) MUC5AC suppressed *P. aeruginosa* PA01 association to plastic and glass surfaces and attachment to live HT human epithelial cells in a concentration dependent manner. **In vivo** **Pig burn wounds:** with MUC5AC 1 week post infection. two-log reductions in *P. aeruginosa* CFUs, no reduction without mucins. Free mucin glycans potently regulate bacterial phenotypes even at relatively low concentrations and regulatory function is likely dependent on glycan complexity, as monosaccharides do not attenuate virulence.
	**Lactobacilli**		
Lenzmeier 2019, USA [31]	10 adult female ND4 Swiss Webster mice given 15% 3rd degree burns, wounds inoculated with *P. aeruginosa strain PA01* (CF687 and CF714) from cystic fibrosis patients in Texas	Lactobacilli strains produce > 48 bacteriocins. Mice received either injection of 20x concentrated supernatant from *Lactobacillus gasseri* strain ATCC 33323 (63 AM) (Lg63AM) [abbreviation = LgCS] after injury and 24 hours post injury, or ceftazidime intra-peritoneally or both. Biofilm formation assessed by confocal laser scanning microscopy	**In vivo** LgCS inhibited the growth of P. aeruginosa strain PAO1, reduced biofilm development 40-fold at 8 hours (control significantly increased) and eliminated biofilms at 28 hours. **In vitro** Mouse burns: local treatment of wound by LgCS did not inhibit *P. aeruginosa* growth in wound at 24 hours but prevented transfer to blood stream with 100% survival of mice at 7 days treated with LgCS (no *P. aeruginosa* in livers or spleens), 100% death due to sepsis in untreated mice (~10^7 cfu/ml *P. aeruginosa* g^{-1} in livers and spleens). Second dose of LgCS 24 hours after first dose completely eliminated *P. aeruginosa* in wound.
	**Phage therapy**		

Alves 2018, UK 33][	Large White or Landrace pigs (~ 10 kg). On the skin (not live pigs) 24 partial thickness 2nd degree burn wounds x 60 seconds, MRSA252-Rif (resistant to rifampicin) (10^4 cfu/ml)	Phages DRA88 and SAB4238-A (10^9 pfu/ml)	**ex-vivo biofilms** 24 hours after phage treatment, phage treated pigs MRSA $10^{6.5}$ cfu/ml compared to control ($10^{7.5}$ cfu/ml) (p ≤ .0001); 48 hours after phage treatment 10^7 cfu/ml compared to control (10^7 cfu/ml) (n.s.); on XTT colorimetric assay at 24 hours phage optical density 0.2 and control 0.7 (p ≤.05); at 48 hours 0.5 and 1 (p ≤.05). **Phage replication** at 24 hours 17.25-fold increase, at 48 hours 64.6-fold increase
Ho 2016, Taiwan [34]	Four ICUs beds in 945 bed teaching hospital, 6 month intervention, standard cleaning with bleach + phage aerosol. 500 ml of 8 phages (10^7 pfu/ml) nebulised into a fine mist of 5-7 µm droplets which saturated each 27 m^3 space in 2.5 minutes, resulting in 5.5 x 10^4 cfu/ml in each room)	8 phages (φAB1, φAB2, φAB6, φAB 7, φ4C08, φ8C07, φAB11, φ 5C05) at	**In vivo** Carbapenem-resistant *Acinetobacter baumanii* (CRAB) ragte = 8.57/1000 patient days pre-intervention, 5.11 during aerosol intervention period (p =.0029), resistant isolates decreased 87.6% to 46.07% (p=.001) **Decreased drug use:** colistin 7,876 DDD/1,000 patient days decreased to 3,158 (p =0.0177); tigecycline 2,737 to 753 (p = .0005); meropenem 5,084 to 2,469 (p=0.0385), imipenem 1,384 to 1,101 (ns).
Holguín 2015, Columbia [35]	(1) Four *P. aeruginosa* strains from Hospital Federico LLeras de Ibagué, Columbia designated P1, P2, P3 and P4 and 4 strains from 4 clinics in Bogotá (10^6 to 10^8 cfu/ml) (2) 5 female Swiss mice with burns with water at 90 °C x 10 seconds in each of 4 phage therapy groups and three control groups	Φ*Pan70* phage (6.5 x 10^7 pfu/ml). P1 and P3 were resistant to ciprofloxacin, gentamicin, imipenem, meropenem, ceftazidime and intermediate resistance to aztreonam and cefepime; P4 resistant to ciprofloxacin, gentamicin, imipenem, meropenem, ceftazidime, intermediate resistance to cefepime and sensitive to aztreonam	**In vitro** at 18 hours after phage therapy *P. aeruginosa* P1 $10^{7.5}$ decreased to 10^4 pfu/ml; P2 10^8 to $10^{4.5}$, P4 $10^{7.5}$ to $10^{2.5}$ (by visual inspection of Figure 2), results for P2 were not reported) **In vitro biofilms** P1 17% reduction at 0 hours (p=.003), 34% at 24 hours (p=.134); 55% at 48 hours (p=.005) P3 59% reduction at 0 hours (p= .00001), 56% at 24 hours (p=.034); 75% at 48 hours (p=.0004) P4 68% reduction at 0 hours (p=.015), 15% at 24 hours (p=.036; 21% at 48 hours (p=.286) **In vivo** Φ*Pan70* given immediately after *P. aeruginosa* infection: 4/5 mice survived; Φ*Pan70* 45 minutes after infection 5/5 survived; 24 and 48 hours after infection 4/5 mice survived; controls all mice died days 3 or 4.
O'Flaherty 2005, Ireland [36]	28 *S. aureus* strains from outpatients, inpatients and hospital staff in Ireland over 3 year period	Phage K ATCC 19685-B1; 14 *S. aureus* strains initially were only weakly sensitive to Phage K but after repeated passage became sensitive to the modified phages	**In vitro** 14/28 *S. aureus* strains sensitive to phage K (10^7 cfu/ml) and no bacteria remained after 2 hours; no bacteriophage-insensitive mutants (BIMs) after 25 hours **In vivo** (1) MRSA strain DPC5645 reduced within 2 hours from 5.7 x 10^6 cfu/ml to undetectable levels (2) MRSA strain DPC5246 on skin reduced 100-fold with phage K (1.4 x 10^8 pfu/ml) (10 replications of experiment, no statement of numbers of participants or hands)
Pallavali 2021, India [37]	*P. aeruginosa* yvu1 (GenBank: KY018605.1). *S. aureus* yvu2 (GenBank: KY496615.1); *K. pneumoniae* yvu3 (GenBank: KY496614.1); and *E. coli* yvu4	Bacteriophages (all at 1 x10^9 pfu/ml) vB_PAnP_PADP4 to infect *P. aeruginosa;*, vB_ECnM_ECDP3 for *E. coli*; vB-KPnM_KPDP1 for *K.*	**In vitro biofilms** Dynamic renewal of media every 12 hours (DR). At 96 hours after 4 hours phage therapy optical density (OD), which corresponds to biomass: (1)

	pneumoniae; and vB_SAnS_SADP1 for *S. aureus*. All assays in triplicate	*P. aeruginosa* 0.47 ± 0.035 decreased to 0.17 ± 0.024 (2) *E. Coli* 0.47 ± 0.035 decreased to 0.15 ± 0.026 (3) *K. pneumoniae* 0.47 ± 0.035 decreased to 0.17 ± 0.022 (4) *S. aureus* 0.47 ± 0.036 decreased to 0.16 ± 0.032 **In vitro confocal microscopy:** Predominant numbers of dead cells after 4 hours phage therapy

MIC = minimum inhibitory concentration of antimicrobial agent; MBIC = minimum inhibitory concentration of antimicrobial agent to inhibit biofilm formation; MBEC = minimum inhibitory concentration of antimicrobial agent to eradicate/degrade mature biofilms.

Pan [27]: Star-shaped polypeptides e.g., polyethylenimine-g-poly(L-ornithine), (PEI-g-PLO), polyethylenimine-g-poly(L-lysine) (PEI-g-PLL) and polyethylenimine-g-poly(L-α,ζ-diaminoheptylic acidCID(pEI-g-PLH.ornithine) consist of a branched PEI core and peripheral chains of PLO, PLL or PLH bearing 3, 4 or 5 methylene groups in their side chains. Two examples are: (1) P03 = (PEI-g-PLO_{20}); (2) PL2 = PEI-g-PLL_{10}); and (3) PH2 = (PEI-g-PLH_{10}), where PEI = polyethylenimine) and PLO = poly(L-ornithine) and PLL= poly(L-lysine).
